# Supplementary material for: Waning of SARS-CoV-2 Seropositivity among Healthy Young Adults over Seven Months
Source: Vaccines (Basel). 2022 Sep 15;10(9):1532. doi: 10.3390/vaccines10091532 (PMC9505545; doi:10.3390/vaccines10091532)
Supplement: Supplementary file 1 [file vaccines-10-01532-s001.zip › vaccines-1891403-supplementary.pdf]

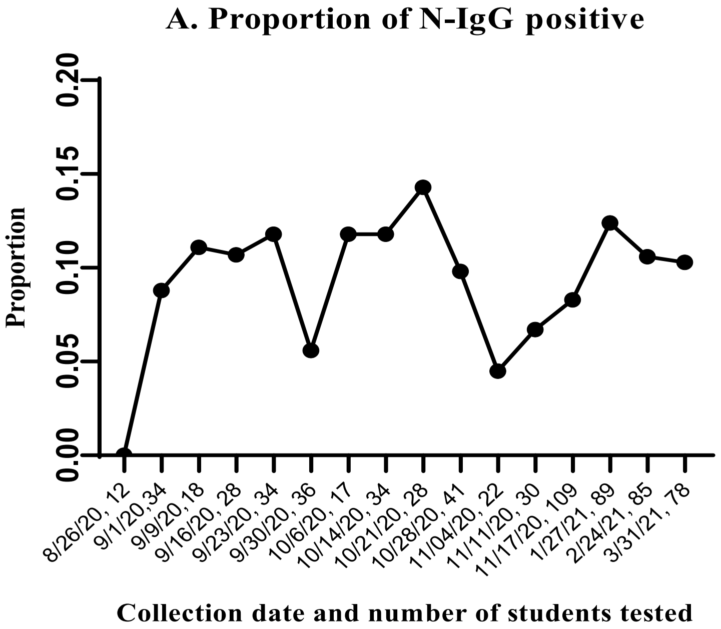

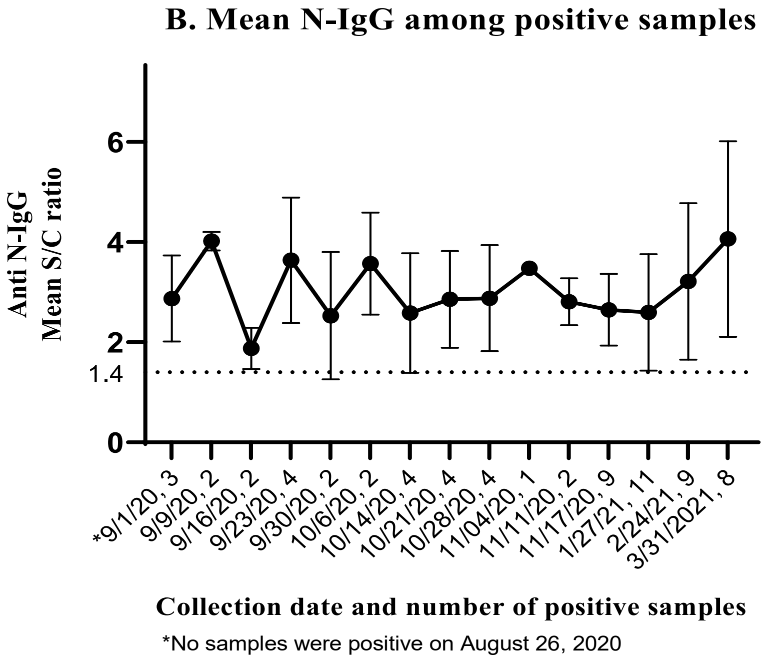

Figure S1. Descriptive Statistics of anti-Nucleocapsid IgG (N-IgG) by date of collection. (**A**) Proportion of tests that were positive among participants tested by collection date. (**B**) Among participants testing N-IgG positive, t he mean S/C ratio is presented by collection date with positive defined as S/C ratio ≥ 1.4.

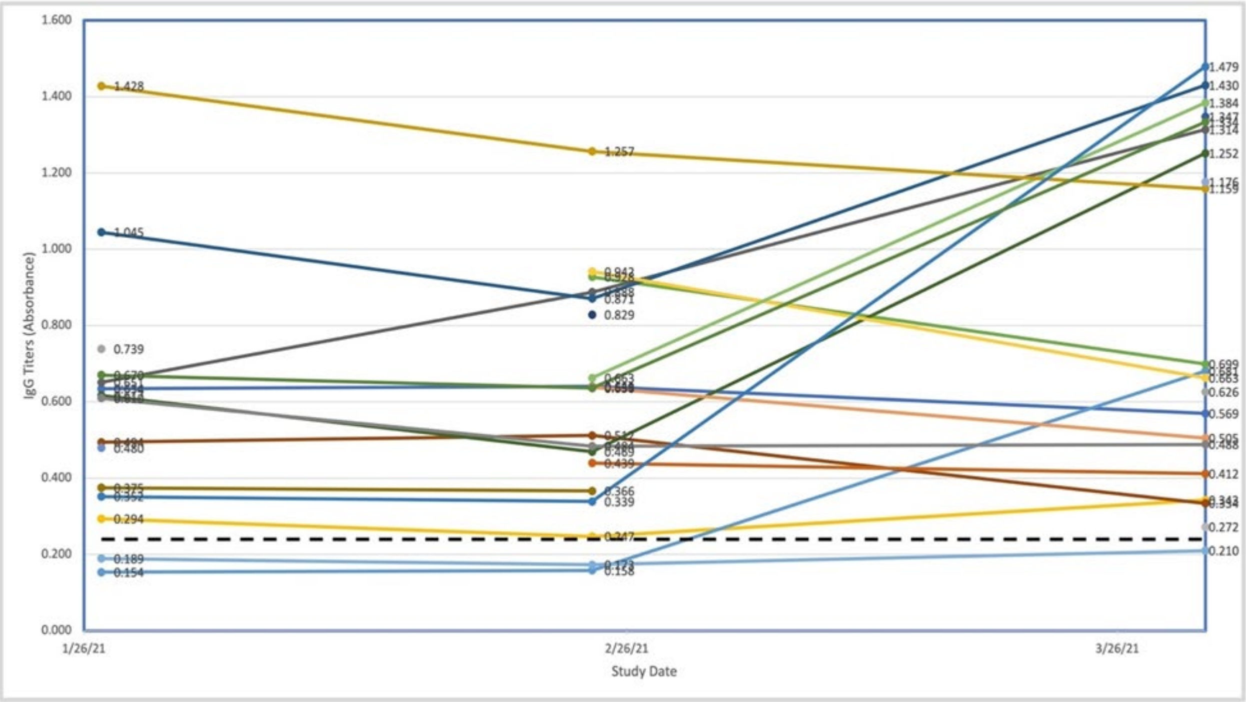

Figure S2. Change in anti-spike protein-IgG for 25 young adults

Legend: Anti Spike-IgG was analyzed using sera collected on January 27, 2021, February 24, 2021, and March 31, 2021. Each color is a unique person. X-axis is date of collection for 25 young adults. Y-axis is ELISA absorbance to anti S-IgG. A positive result was defined as four-fold the negative control value (0.06), or 0.24 optical density absorbance. On March 31, 2021, vaccinated participants had the following S-IgG values, dose-vaccination date: 0.343, Dose 1-3/27/21; .0681, Dose 2- 3/30/21; 0.334, Dose 1- 3/31/21; 1.314, Dose 1- 3/20/21; 1.430, Dose 1- 3/19/21; 1.252, Dose 1- 3/25/21; 1.384, Dose 1- 3/11/21; 1.347, Dose 1&2- 3/3/21 & 3/30/21; 1.479, Dose 1- 3/5/21; 1.334, Dose 1- 3/14/21; 1.176, Dose 1&2- 1/27/21 & 2/17/21.

Table S1. Characteristics of Study Population by Nucleocapsid IgG (N-IgG) Status

	Ever N-IgG Positive		Always N-IgG Negative	
	Number	%*	Number	%
Age				
18 - 20	20	71.43	43	39.81
21- 22	6	21.43	45	41.67
23 -25	1	3.57	12	11.11
26+	1	3.57	8	7.41
Total	28	100	108	
Biologic sex				
Male	9	32.14	33	30.56
Female	19	67.86	75	69.44
Total	28	100	108	
Race				
White/Caucasian	21	75	86	78.7
Black/African American	3	10.71	8	7.41
Asian	1	3.57	7	6.48
Other	3	10.71	2	1.85
Total	28	100	6	5.56
Ethnicity				
Latinx	6	22.22	10	9.43
not Latinx	21	77.78	96	90.57
Total	27	100	106	
School Year, fall 2020				
Freshman	9	32.14	19	17.59
Sophomore	6	21.43	7	6.48
Junior	6	21.43	19	17.59
Senior	5	17.86	43	39.81
Graduate School	2	7.14	20	18.52
	28	100	108	
Had a COVID-19 Test before enrollment				
No	6	21.43	52	48.15
Yes	22	78.57	55	50.93
Do not know	0	0	1	0.93
Total	28	100		
COVID-19 Vaccine as of 3/31/21**				
First dose	11	39.3	28	40.58
Second dose	3	10.71	17	24.64

No dose of vaccine	14	50	24	34.78
Total	28	100	69	100
Tested COVID-19 positive before enrollment				
Positive	8	28.57	2	0.02
Negative	13	46.43	52	48.15
Waiting on result	0	0.00	1	0.01
Inconclusive	1	0.04	0	0.00
missing	6	21.43	53	49.10
	28		108	
Flu Vaccine 2019-2020 season				
No	14	50	36	33.33
Yes	11	39.29	65	60.19
Do not know	3	10.71	7	6.48
	28		105	

* Some percentages do not add to 100 due to missing values.
** Denominator is 97; vaccine offered in spring semester 2021

Table S2. Descriptive statistics of anti-Spike IgG by date of collection and vaccination status

				Vaccinated	Not Vaccinated
	1/27/2021	2/24/2021	3/31/2021	3/31/2021	3/31/2021
Number of values	15	20	21	11	10
Minimum	0.154	0.158	0.21	0.334	0.21
25% Percentile	0.352	0.3843	0.45	0.681	0.377
Median	0.61	0.637	0.681	1.314	0.537
75% Percentile	0.67	0.8605	1.324	1.384	0.672
Maximum	1.428	1.257	1.479	1.479	1.159
Range	1.274	1.099	1.269	1.145	0.949
95% CI of median					
Actual confidence level	96.48%	95.86%	97.34%	98.83%	97.85%
Lower confidence limit	0.352	0.439	0.488	0.343	0.272
Upper confidence limit	0.67	0.829	1.314	1.43	0.699
Mean	0.5821	0.606	0.8418 *	1.098 **	0.5603
Std. Deviation	0.328	0.2869	0.4472	0.4312	0.2643
Std. Error of Mean	0.0847	0.06415	0.09759	0.13	0.08358
Lower 95% CI of mean	0.4005	0.4717	0.6382	0.808	0.3712
Upper 95% CI of mean	0.7638	0.7402	1.045	1.387	0.7494
Geometric mean	0.5002	0.5322	0.7154	0.9792	0.5064
Geometric SD factor	1.809	1.753	1.853	1.761	1.626
Lower 95% CI of geo. mean	0.3603	0.4092	0.5403	0.6696	0.3577
Upper 95% CI of geo. mean	0.6945	0.6921	0.9471	1.432	0.717

* mean significantly different from Spike IgG value on 2/24/21, unpaired *t*-test, $p = 0.053$

** mean significant different from unvaccinated Spike IgG value on 3/31/21, unpaired *t*-test, $p = 0.003$
